# Magnetite nanoparticles−based hydroxyl radical scavenging activity assay of antioxidants using N, N-dimethyl-p-phenylenediamine probe

**DOI:** 10.3906/kim-2006-9

**Published:** 2020-10-26

**Authors:** Ziya CAN, Büşra KESKİN, Ayşem ARDA, Erol ERÇAĞ, Mustafa Reşat APAK

**Affiliations:** 1 Department of Chemistry, Faculty of Engineering, İstanbul University-Cerrahpaşa, İstanbul Turkey; 2 Department of Chemistry, Institute of Graduate Studies, İstanbul University-Cerrahpaşa Turkey; 3 Department of Chemistry, Faculty of Arts and Sciences, Tekirdağ Namık Kemal University, Tekirdağ Turkey; 4 Turkish Academy of Sciences (TÜBA), Ankara Turkey

**Keywords:** Magnetite (Fe _3_ O _4_ ) nanoparticles, reactive oxygen species (ROS), N, N-Dimethyl-p-phenylenediamine, colorimetric probe, antioxidant activity

## Abstract

Excessive amounts of reactive oxygen species (ROS), unless counterbalanced by antioxidants, can cause cellular damage under oxidative stress conditions; therefore, antioxidative defenses against ROS must be measured. With the development of nanotechnology, nanoparticles have found numerous applications in science, health, and industries. Magnetite nanoparticles (Fe _3_ O _4_ :MNPs) have attracted attention because of their peroxidase-like activity. In this study, hydroxyl radicals (•OH) generated by MNPs-catalyzed degradation of H _2_ O _2_ converted the N,N-dimethyl-p-phenylenediamine (DMPD) probe into its colored DMPD•+ radical cation, which gave an absorbance maximum at λ = 553 nm. In the presence of antioxidants, •OH was partly scavenged by antioxidants and produced less DMPD• ^+^ , causing a decrease in the 553 nm-absorbance. Antioxidant concentrations were calculated with the aid of absorbance differences between the reference and sample solutions. The linear working ranges and trolox equivalent antioxidant capacity coefficients of different classes of antioxidants were determined by applying the developed method. In addition, binary and ternary mixtures of antioxidants were tested to observe the additivity of absorbances of mixture constituents. The method was applied to real samples such as orange juice and green tea. Student t-test, F tests, and the Spearman’s rank correlation coefficient were used for statistical comparisons.

## 1. Introduction

Reactive oxygen species (ROS) is a collective term expressing both radicalic oxygen species [e.g., hydroxyl (•OH) and superoxide radicals (O _2_ •−)] together with other nonradical derivatives of oxygen, such as, singlet oxygen ( ^1^ O _2_ ) and hydrogen peroxide (H _2_ O _2_ ). Due to beneficial and harmful health effects, the amount of ROS in the body plays a central role in biological processes. Oxidative damage of ROS to cells and biomolecules [1,2] may be compensated for by intrinsic and extrinsic antioxidants. If the delicate balance between ROS generation and antioxidative defenses of the organism is not conserved, oxidative stress conditions may occur [1]. Oxidative stress is related to the general aging process and cell death, affecting all organ systems [3]. ROS also plays a part in many age-related diseases, such as Alzheimer [4], Parkinson [5], and cardiovascular diseases [6]. Among all ROS, •OH is considered as one of the most reactive species, because it can rapidly attack biomolecules with a high oxidation potential and cause irreversible damage [7]. Therefore, the measurement of ROS scavenging activity of different classes of antioxidants is important. Quantitative analysis of total antioxidant activity can be carried out using globally adopted molecular spectroscopic methods. Among the free radical scavenging assays, those using artificial radicals such as ABTS (2,2’-azinobis (3-ethylbenzothiazoline-6-sulfonic acid) [8,9] and DPPH (2,2-diphenyl-1-picrylhydrazyl) [10, 11], or ORAC as the peroxyl radical scavenging method (Oxygen Radical Absorbance Capacity) [12,13] have emerged as the most popular ones. The transition metal ion reducing capacity of antioxidants can be measured with CUPRAC (Cupric Reducing Antioxidant Capacity) [14,15] and FRAP (Ferric Ion Reducing Antioxidant Power) assays [16-19]. Many alternative spectrophotometric [20–23] and voltammetric methods [24,25] were later developed, based on different mechanisms of antioxidant and radical scavenging action. With the advancement of nanoscience, many new methods are on the rise due to extraordinary physico-chemical properties of nanoparticles, such as large surface-to-volume ratio for enhanced catalysis and surface plasmon resonance absorption giving rise to high sensitivity.

The peroxidase-like activity of MNPs has already been established [26] and applied to many different areas such as the removal of organic pollutants [27,28], hydrogen peroxide [29–31], and analysis of home-made explosives (e.g., TATP) [32]. The peroxidase-like activity of Fe _3_ O _4_ NPs is based on the simultaneous existence of Fe ^2+^ /Fe ^3+^ ion pair in the structure, having a similarity to Fenton’s reagent [26].

In the developed method, hydroxyl radicals were produced through MNPs-catalyzed degradation of H _2_ O _2_ , owing to the peroxidase-like activity of Fe _3_ O _4_ . Since a part of the obtained hydroxyl radicals were scavenged with antioxidants, the remaining •OH could oxidize DMPD to the colored DMPD+ radical cation. Both the reference (the solution containing no antioxidant) and samples were measured against water at 553 nm. For the calculations, the difference between the reference and sample absorbances (ΔA) was used. It is better to monitor a Fenton-type reaction of •OH generation with MNPs because Fe _3_ O _4_ can be magnetically separated at any stage while soluble Fe(II)-catalyzed degradation of H _2_ O _2_ in homogenous solution is less controllable. The developed method was applied to different classes of antioxidants and their binary/ternary mixtures. In addition, it was applied to real samples such as, orange juice and green tea, and its statistical comparisons were made with the aid of Student t-test, F test, and Spearman’s rank correlation tests.

## 2. Materials and methods

### 2.1. Instrumentation and chemicals

Shimadzu UV-1800 (Kyoto, Japon) spectrophotometer was used for absorption measurements. Hellma GmbH & Co. KG (Müllheim, Germany) quartz cuvettes with 1 cm optical thickness were used for the UV-Vis measurements.

All reagents were of analytical reagent grade unless otherwise stated. Quercetin (QR), gallic acid (GA), ferulic acid (FA), caffeic acid (CFA), catechin (CAT), epicatechin, trolox (TR), para-coumaric acid (p-CUM), L-ascorbic acid (AA), L-cysteine (CYS), morin (MR), rutin (RT), hydrogen peroxide (30% by mass), and N,N-Dimethyl-p-phenylenediamine (DMPD) were purchased from Sigma-Aldrich Chemie GmbH (Steinheim, Germany), and all other reagents from Merck KGaA (Darmstadt, Germany) and Sigma-Aldrich Chemie GmbH. Commercial orange juice and green tea samples were obtained from local markets.

### 2.2. Preparation of solutions

Except for the ascorbic acid, all antioxidant solutions were prepared in ethanol. Ascorbic acid, mixtures containing AA, and diluted orange juice samples were prepared in 1:1 (v/v) ethanol:water.

H _2_ O _2_ solution (4 × 10 ^-4^ mol L ^-1^ ), pH 3.6 acetic acid buffer solution (containing a total CH _3_ COOH/CH _3_ COONa concentration of 2 mol L ^-1^ ), Fe _3_ O _4_ NPs solution (400 mg L ^-1^ ), and N,N-dimethyl-p-phenylenediamine (DMPD) solution (2 × 10 ^-3^ mol L ^-1^ ) were prepared in ultrapure water.

To prepare the green tea sample, 50 mL boiled water was added to 0.5 g of green tea and let to stand for 15 min for infusion [25]. Then, the final solution was passed through filter paper and diluted to 50 mL with ultrapure water. This stock solution was appropriately diluted and the proposed method was applied.

For the orange juice sample, orange juice was centrifuged at 10000 rpm for 20 min and the supernatant filtered with the aid of a microfilter. The proposed method was applied to this sample with appropriate dilution. The results of real samples were given in trolox equivalents (TE).

### 2.3. Standard addition of AA to orange juice

To determine the recovery of AA from orange juice samples, the standard addition method was applied. The analyses of 1:200 diluted orange juice sample, 2 × 10 ^-5^ mol L ^-1^ AA solution at final concentration, and AA-added solution were carried out separately with the proposed method.

### 2.4. Synthesis of Fe _3_ O _4_ NPs

The coprecipitation method was carried out for synthesizing Fe _3_ O _4_ NPs [29]. In the beginning, 10 mL of FeCl _2_ (2 mol L ^-1^ ) solution in 2 mol L ^-1^ HCl were mixed with 50 mL of FeCl3 (1 mol L ^-1^ ) solution. After deoxygenation with nitrogen gas, the mixture solution was added drop wise to 500 mL of oxygen-free aqueous ammonia solution (0.7mol L ^-1^ ). The final solution was stirred under a nitrogen atmosphere for 30 min. Fe _3_ O _4_ NPs were separated and washed with water.

### 2.5. Application of the proposed method

One milliliter of 4 × 10 ^-4^ mol L ^-1^ H _2_ O _2_ solution, 1 mL of pH 3.6 acetate buffer, 1 mL of 400 mg L ^-1^ Fe _3_ O _4_ NPs solution, 1 mL of antioxidant solution (for reference solution, depending on the solvent of the sample, EtOH or 1:1 (v:v) EtOH: water was used), and 1 mL of 2 × 10 ^-3^ mol L ^-1^ DMPD solution were mixed in this order. Finally, at the end of the 30 min, the absorbances of all solutions (including the reference solution) were measured against water at 553 nm. Antioxidant activity was expressed as the decrease of absorbance in the presence of scavengers.

### 2.6. Statistical analysis

CFA and RT were analyzed by the thiobarbituric acid reactive substances (TBARS) method, based on colored product formation of TBA with the peroxidation end product malondialdehyde [20]. CFA and RT solutions were performed with the proposed method at 4 × 10 ^-6^ and 2 × 10 ^-4^ mol L ^-1^ , and with TBARS method at 4 × 10 ^-6^ and 2 × 10 ^-5^ mol L ^-1^ , respectively. Rutin results, which were obtained by TBARS method, were multiplied by the dilution factor (10) for correction of concentration. The obtained results from both methods were used for statistical comparison using Student t-test and F tests. Also, Spearman’s rank correlation (using Equation 1) was used for statistical comparison of ROS scavenging activity of selected antioxidants [33].

(1)rs=1-6∑i2dn(n2-1)

r _s_ = Spearman’s rank correlation,

d _i_ = Difference between two ranks,

n =Number of pairs.

## 3. Results

**Figure 1:**
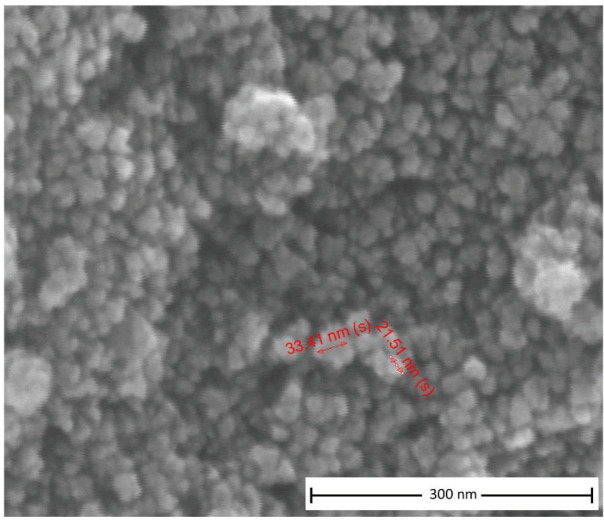
SEM image of 20–30 nm sized Fe _3_ O _4_ particles.

When scanning electron microscopy (SEM) analysis was performed to observe the shape and size of synthesized magnetite nanoparticles, they were found to be spherical and in between 20 and 30 nm in diameter (Figure 1). It is known from the literature that smaller size magnetite nanoparticles show higher catalytic activity [26]. Magnetic properties of magnetite nanoparticles were also observed by applying a magnetic field with a magnet.

The proposed method is based on the production of ROS (i.e. mainly hydroxyl and perhydroxyl radicals) from H _2_ O _2_ with the aid of Fe _3_ O _4_ NPs at pH 3.6, scavenging of produced ROS by antioxidants, and finally oxidation by remaining ROS of the DMPD probe to the pink colored DMPD ^•+^ radical cation (Scheme). Spectrophotometric measurements were performed with this obtained colored product.

**Scheme:**
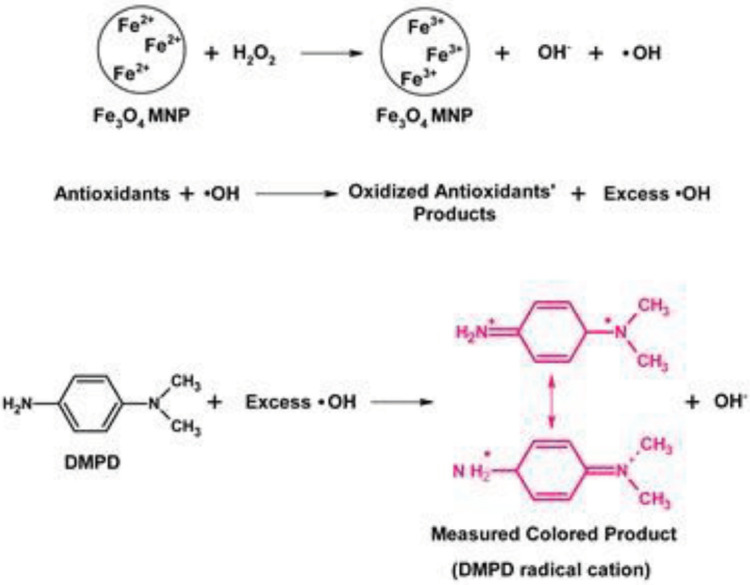
Schematic presentation of ROS scavenging activity of antioxidants with the proposed method.

### 3.1. Optimization of reaction parameters

**Figure 2:**
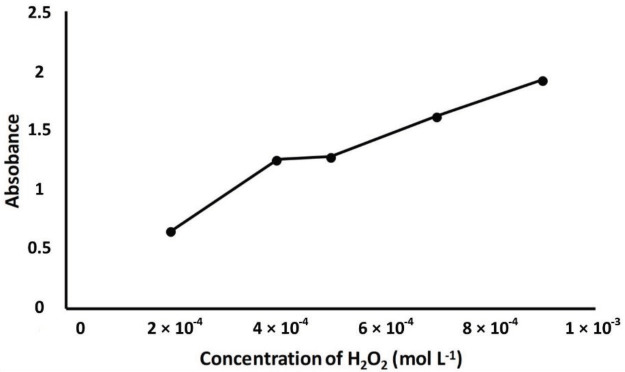
Effect of H _2_ O _2_ concentration on DMPD•+ absorbance. Working conditions: 1 mL of H _2_ O _2_ + 1 mL of pH 3.6 acetic acid buffer + 1 mL of 400 mg L ^-1^ Fe _3_ O _4_ NPs + 1 mL of ethanol (wait for 5 min) + 1 mL of 5 × 10 ^-3^ mol L ^-1^ DMPD (wait for 30 min) + centrifuge (for 5 min at 5000 rpm). All measurements were made against water at 553 nm.

**Figure 3:**
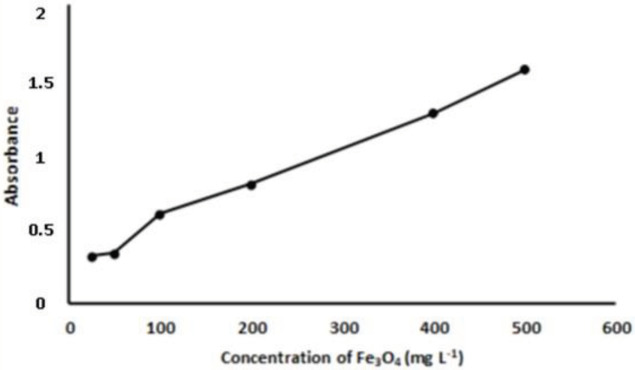
Effect of F _3_ O _4_ NPs concentration on DMPD ^•+^ absorbance. Working conditions: 1 mL of 4 × 10 ^–4^ mol L ^-1^ H _2_ O _2_ + 1 mL of pH 3.6 acetic acid buffer + 1 mL of Fe _3_ O _4_ NPs + 1 mL of ethanol (wait for 5 min) + 1 mL of 5 × 10 ^-3^ mol L ^-1^ DMPD (wait for 30 min) + centrifuge (for 5 min at 5000 rpm). All measurements were made against water at 553 nm.

**Figure 4:**
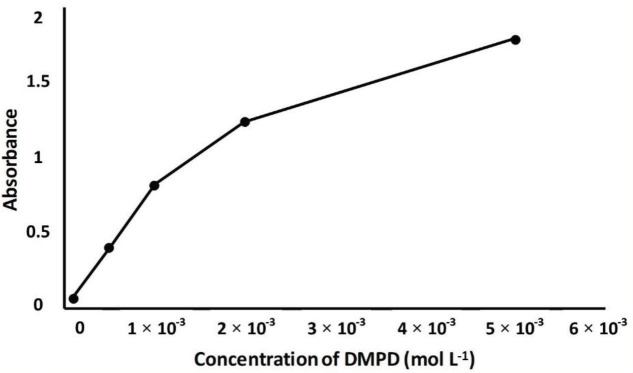
Effect of DMPD concentration on absorbance. Working conditions: 1 mL of 4 × 10 ^–4^ mol L ^-1^ H _2_ O _2_ + 1 mL of pH 3.6 acetic acid buffer + 1 mL of 400 mg L ^-1^ Fe _3_ O _4_ NPs + 1 mL of ethanol (wait for 5 min) + 1 mL of DMPD (wait for 30 min) + centrifuge (for 5 min at 5000 rpm). All measurements were made against water at 553 nm.

For optimization of the developed method, H _2_ O _2_ , Fe _3_ O _4_ NPs, and DMPD concentrations were investigated. As shown in Figures 2–4, for increased concentrations of substances, increasing absorbance values were obtained. For selecting the appropriate concentrations of reagents, attention was paid to the absorbance value of the reference solution which should lie in the range of 1.1–1.2. According to this precaution, H _2_ O _2_ concentration was determined as 4 × 10 ^-3^ mol L ^-1^ , Fe _3_ O _4_ NPs concentration as 400 mg L ^-1^ , and DMPD concentration as 2 × 10 ^-3^ mol L ^-1^ . For preserving the solubility and stability of magnetite nanoparticles, the selection of an appropriate working pH is important. In this work, Fe _3_ O _4_ nanoparticles were used as a catalyst in conjunction with DMPD sensing. Without deactivating the catalyst and without allowing ferric iron to hydrolyze, an optimal pH of 3.6 was selected, as in the literature [34,35].

### 3.2. Determination of hydroxyl radical scavenging activity of antioxidants with the aid of Fe _3_ O _4_ NPs and DMPD probe

**Figure 5:**
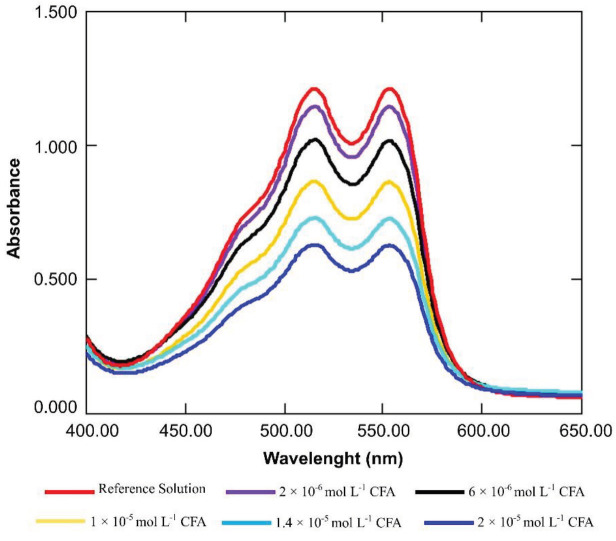
The DMPD•+ spectra showing the •OH scavenging activity of different concentrations of caffeic acid solutions.

The developed method was applied to CYS, AA, TR, and different classes of antioxidants such as flavonol (QR, MR), phenolic acid (GA, FA, CFA, p-CUM), flavanol (CAT, EC), and flavon (RT). The calibration curves were drawn between antioxidant concentrations and absorbance decrements (ΔA). When the developed method was applied to caffeic acid, the obtained DMPD•+ spectra are shown in Figure 5, indicating absorbance decrements with increasing CFA concentration. Two peaks were observed at 553 and 515 nm, with quite close absorbance values. The 553 nm-peak was preferred for further measurements so that it would be less affected by plant pigments absorbing in the visible range.

The calibration equations, linear ranges of antioxidant concentration, trolox equivalent antioxidant capacity (TEAC) values, and correlation coefficients are shown in Table 1. According to the developed method, hydroxyl radical scavenging efficiency of tested antioxidants followed the order: EC > CFA > FA > GA > CAT >CYS > QR > MR > AA > p-CUM > RT. This finding complies with the hypothesis of Bors and Michel based on experimental results of pulse radiolysis transient spectra and decay rate constants of •OH-absorbed species that catechol and/or pyrogallol groups were the active sites of radical attack, with subsequent conversion to quinoid compounds [36]. In this research, catechins, CFA, and GA with o-dihydroxy phenol (catechol) substitution were the leading •OH scavengers, in accordance with the findings of Demirci Çekiç et al. [21] who demonstrated the priority of o-catechol and galloyl moieties of phenolic antioxidants in hydroxyl radical scavenging ability measured with a DMPD probe on a Nafion membrane.

**Table 1:** The linear concentration ranges, calibration equations, and TEAC coefficients of the tested antioxidants in the proposed method.

Antioxidant	Linear range (Final concentrations) (mol L ^-1^ )	Calibration equations and TEAC values
Trolox (TR)	2×10 ^-5^ – 1.2×10 ^-4^	ΔA = 1.05 × 10 ^4^ C – 0.04 (r = 0.9966)
Quercetin (QR)	2×10 ^-5^ – 1×10 ^-4^	ΔA = 9.96 × 10 ^3^ C – 0.03 (r = 0.9981) (TEAC Coeff. = 0.95)
Gallic acid (GA)	2×10 ^-6^ – 2×10 ^-5^	ΔA = 2.07 × 10 ^4^ C + 0.14 (r = 0.9976) (TEAC Coeff. = 1.97)
Ferulic acid (FA)	2×10 ^–6^ – 2×10 ^-5^	ΔA = 2.54 × 10 ^4^ C + 0.03 (r = 0.9966) (TEAC Coeff. = 2.42)
Caffeic acid (CFA)	2×10 ^-6^ – 2×10 ^-5^	ΔA = 3.05×10 ^4^ C + 0.03 (r = 0.9977) (TEAC Coeff. = 2.90)
Catechin (CAT)	2×10 ^-6^ – 2×10 ^-5^	ΔA = 1.7 × 104C + 0.09 (r = 0.9992)(TEAC Coeff. = 1.62)
Epicatechin (EC)	2×10 ^-6^ – 2×10 ^-5^	ΔA = 3.12 × 104C + 0.04 (r = 0.9982)(TEAC Coeff. = 2.97)
p-coumaric acid (p-CUM)	1×10 ^-4^ – 1×10 ^-3^	ΔA = 6.56 × 10 ^2^ C + 0.28 (r = 0.9917) (TEAC Coeff. = 0.06)
Ascorbic acid (AA)	2×10 ^-5^ – 2×10 ^-4^	ΔA = 2.01 × 10 ^3^ C + 0.08 (r = 0.9996) (TEAC Coeff. = 0.19)
L-cysteine (CYS)	1×10 ^-5^ – 4×10 ^-5^	ΔA = 1.53 × 10 ^4^ C + 0.09 (r = 0.9948) (TEAC Coeff. = 1.46)
Morin (MR)	1.2×10 ^-5^ – 2×10 ^-4^	ΔA = 6.28 × 10 ^3^ C + 0.08 (r = 0.9951) (TEAC Coeff.= 0.6)
Rutin (RT)	1×10 ^-4^ – 1×10 ^-3^	ΔA = 3.29 × 10 ^2^ C + 0.16 (r = 0.9979) (TEAC Coeff. = 0.03)

### 3.3. Analysis of synthetic antioxidant mixtures and real samples

When the additivity of differential absorbances of antioxidants in their binary and ternary synthetic mixtures was tested with the proposed method, the percentage relative errors (calculated from the difference of expected and found values) of mixtures ranged between –7 and +5% (Table 2). This basically showed the absence of chemical deviations from Beer’s law in the proposed method.

As real samples, orange juice and green tea were analyzed with the proposed method, and the results were calculated in trolox equivalents (TE) as defined in the literature [24,25]. The antioxidant capacity of orange juice, and green tea were found as 2.33 mmol TE L ^-1^ , and 1.23 mmol TE g–1, respectively. One juice sample was also spiked with an antioxidant compound to observe recovery and to confirm the absence of chemical deviations from Beer’s law in a real sample. Standard addition of a certain concentration of 2 × 10 ^-5^ mol L ^-1^ AA solution to diluted orange juice yielded an AA recovery of 97% with the proposed method.

**Table 2:** Relative errors of binary and ternary synthetic antioxidant mixtures.

Antioxidant mixtures (in final conc.)	Relative errors (%)
5 × 10 ^-6^ mol L ^-1^ CAT + 6 × 10 ^-6^ mol L ^-1^ FA	–2.00
5 × 10 ^-6^ mol L ^-1^ CAT + 4 × 10 ^-5^ mol L ^-1^ QR	–0.04
5 × 10 ^-6^ mol L ^-1^ CAT + 4 × 10 ^-5^ mol L ^-1^ Morin	–1.00
5 × 10 ^-5^ mol L ^-1^ AA + 2 × 10 ^-5^ mol L ^-1^ GA	–3.00
5 × 10 ^-5^ mol L ^-1^ AA + 6 × 10 ^-6^ mol L ^-1^ FA	–3.00
5 × 10 ^-5^ mol L ^-1^ AA + 4 × 10 ^-5^ mol L ^-1^ QR	–7.00
1 × 10 ^-6^ mol L ^-1^ CFA + 1 × 10 ^-5^ mol L ^-1^ QR + 1 × 10 ^-6^ mol L ^-1^ CAT	–6.00
1 × 10 ^-5^ mol L ^-1^ TR + 1 × 10 ^-6^ mol L ^-1^ CAT + 1 × 10 ^-6^ mol L ^-1^ GA	+5.00
2 × 10 ^-5^ mol L ^-1^ AA + 2 × 10 ^-5^ mol L ^-1^ TR + 2 × 10 ^-6^ mol L ^-1^ CFA	–6.00

### 3.4. Statistical evaluation of the developed method

For method validation, reference TBARS method was applied to CFA and RT solutions [20]. The CFA and RT solutions in ethanol at 1.0×10 ^-6^ – 5.0×10 ^-6^ mol L ^-1^ concentrations were measured and calibration curves were obtained. A CFA solution at 4 × 10 ^-6^ mol L ^-1^ concentration was tested using both the developed and TBARS methods. For method validation with RT, a solution at 2 × 10-4 mol L ^-1^ concentration was used for the developed method and 2 × 10-5 mol L ^-1^ concentration was used for TBARS method, and then the calculated result was multiplied by the dilution factor. The measurements were carried out for N = 5 repetitive analyses. The compared methods showed no significant differences between the precision and accuracy of results found for CFA and RT (Table 3). The Student t-test and F tests were used for statistical comparison of the population means and variances, respectively, at 95% confidence levels for both tests (Table 3).

For comparing the proposed method with ABTS (radical scavenging) reference assay using the TEAC values of FA, GA, CAT, QR, MR, AA, p-CUM, and RT, Spearman’s rank correlation was used (Table 4). The results showed a ‘strong’ (Spearman’s rank correlation coefficient, rs = 0.78571) relationship between the developed method and ABTS assay (P = 0.02082; two-tailed).

**Table 3:** Statistical comparison of the developed method with reference TBARS method for CFA and RT solutions with the aid of Student t-test and F tests.

Analyte	Method	Mean conc.(mol L ^-1^ )	SD (σ)	S ^a,b^	t ^a,b^	t _table_^b^	F ^b^	F _table_^b^
CFA	Developed method	4.30 × 10 ^-6^	2.27 × 10 ^-7^	-	-	-	-	-
	TBARS method	4.14 × 10 ^-6^	2.27 × 10 ^-7^	3.08 × 10 ^-7^	0.846	2.306	0.371	6.39
RT	Developed Method	2.10 × 10 ^-4^	3.18 × 10 ^-5^	-	-	-	-	-
	TBARS Method	2.17 × 10 ^-4^	2.22 ×10 ^-5^	2.74 × 10 ^-5^	0.415	2.306	2.045	6.39

^a^ S ^2^ = {(n _1_ – 1)s _1_^2^ + (n _2_ – 1)s _2_^2^ }/(n _1_ + n _2_ – 2) and t = (ā _1_ – ā _2_ )/{S(1/n _1_ + 1/n _2_ ) ^1/2^ }, where S is the pooled standard deviation, s _1_ and s _2_ are the standard deviations of the two populations with sample sizes of n _1_ and n _2_ , and sample means of ā _1_ and ā _2_ respectively (t has (n _1_ + n _2_ – 2) degrees of freedom); here, n _1_ = n _2_ = 5. ^b^ Statistical comparison made on paired data produced with proposed and reference methods; the results given only on the row of the reference method.

**Table 4:** Spearman’s rank correlation analysis according to TEAC values of antioxidants.

	1	2	3	4	5	6	7	8	rs	Definition
Developed method	FA	GA	CAT	QR	MR	AA	p-CUM	RT	0.78571	Strong
ABTS/persulfate	GA	CAT	QR	FA	MR	p-CUM	RT	AA		

## 4. Discussion

A novel easy and low-cost DMPD-based ROS scavenging activity method was developed with the aid of Fe _3_ O _4_ NPs having peroxidase-like activity. MNPs catalyzed the degradation of H _2_ O _2_ to ROS (mainly hydroxyl radicals) which in turn converted DMPD to a colored radical cation, and antioxidants (when present) quenched •OH, thereby causing a decrement in the DMPD•+ absorbance. Although a Fenton-type reaction (catalyzed by soluble Fe ^2+^ ions) in homogeneous solution can also generate •OH from H _2_ O _2_ , the MNPs used in this work enable a more easily monitored reaction as the reaction can be stopped at any stage by magnetic separation of Fe _3_ O _4_ NPs. ROS scavenging activity of different classes of antioxidants was determined with the developed method. Polyphenol compounds are attacked by hydroxyl radical predominantly at the o-dihydroxy site [37]; this can be explained by the formation of stable semiquinone radicals (i.e. stabilized by intramolecular hydrogen bonding) from the reaction of the o-dihydroxy group with hydroxyl radicals [38]. Inspection of the most effective hydroxyl radical scavengers of this work, i.e. EC > CFA > FA > GA > CAT, reveals that all these compounds have the 3,4-dihydroxyphenyl (FA having 4-hydroxy-3-methoxyphenyl) moiety that can be attacked by •OH to produce stable semiquinone radicals. Likewise, the cell protective activity of quercetin against oxidative hazard was attributed to certain structure-activity relationships including the o-dihydroxy structure in the B-ring [39]. Also, binary and ternary synthetic antioxidant mixtures were measured and the additivity of antioxidant concentrations (in trolox equivalents) was observed. In addition, the developed method was successfully applied to the real samples of green tea and orange juice. The proposed method was validated against the TBARS method through analysis of CFA and RT standard solutions. Spearman’s rank correlation (tested on 8 antioxidants) was found ‘strong’ between the developed method and the ABTS (radical scavenging) assay.
